# Biosafety evaluation of culture-expanded human chondrocytes with growth factor cocktail: a preclinical study

**DOI:** 10.1038/s41598-020-78395-y

**Published:** 2020-12-09

**Authors:** Maimonah-Eissa Al-Masawa, Wan Safwani Wan Kamarul Zaman, Kien-Hui Chua

**Affiliations:** 1 Department of Physiology, Faculty of Medicine, Universiti Kebangsaan Malaysia Medical Centre, Jalan Yaacob Latiff, Bandar Tun Razak, Cheras, 56000 Kuala Lumpur, Malaysia; 2 Department of Biomedical Engineering, Faculty of Engineering, University of Malaya, 50603 Kuala Lumpur, Malaysia

**Keywords:** Biotechnology, Cell biology, Molecular biology, Physiology

## Abstract

The scarcity of chondrocytes is a major challenge for cartilage tissue engineering. Monolayer expansion is necessary to amplify the limited number of chondrocytes needed for clinical application. Growth factors are often added to improve monolayer culture conditions, promoting proliferation, and enhancing chondrogenesis. Limited knowledge on the biosafety of the cell products manipulated with growth factors in culture has driven this study to evaluate the impact of growth factor cocktail supplements in chondrocyte culture medium on chondrocyte genetic stability and tumorigenicity. The growth factors were basic fibroblast growth factor (b-FGF), transforming growth factor β2 (TGF β2), insulin-like growth factor 1 (IGF-1), insulin-transferrin-selenium (ITS), and platelet-derived growth factor (PD-GF). Nasal septal chondrocytes cultured in growth factor cocktail exhibited a significantly high proliferative capacity. Comet assay revealed no significant DNA damage. Flow cytometry showed chondrocytes were mostly at G0-G1 phase, exhibiting normal cell cycle profile with no aneuploidy. We observed a decreased tumour suppressor genes’ expression (p53, p21, pRB) and no *TP53* mutations or tumour formation after 6 months of implantation in nude mice. Our data suggest growth factor cocktail has a low risk of inducing genotoxic and tumorigenic effects on chondrocytes up to passage 6 with 16.6 population doublings. This preclinical tumorigenicity and genetic instability evaluation is crucial for further clinical works.

## Introduction

Adult cartilage has a limited regenerative capability due to its unique structure as it is avascular, aneural, and alymphatic^1^. Autologous chondrocyte implantation (ACI) and matrix-assisted autologous chondrocyte implantation (M-ACI) are the most promising cell-based treatments to treat cartilage defects^2–4^. In both procedures, chondrocytes are retrieved from patients’ own cartilage, serially expanded in vitro, and re-implanted into the patients’ defective site^2,5^. This expansion procedure is essential to augment the limited number of isolated chondrocytes to generate a clinical size graft while shortening the waiting time for patients before implantation. However, culture-expanded chondrocytes tend to lose their chondrogenic phenotype, resembling fibroblasts. This is manifested by reducing the production of cartilage-specific constituents such as collagen type II, aggrecan, and proteoglycan; while raising production of non-specific cartilage constituents such as collagen type I in a process called dedifferentiation^6,7^. Dedifferentiation is also characterised by decreased synthesis of growth factors, decreased cell proliferation, increased apoptosis, and cell senescence^7,8^. Neocartilage formed by dedifferentiated chondrocytes has been reported to be structurally and functionally inferior to the normal tissue^9,10^. Dedifferentiation was found to elevate the postoperative failure rate in patients after ACI^11^. Of the strategies employed to improve chondrocyte culture condition is the supplementation of culture medium with cartilaginous growth factors^12^. The selection of these growth factors is based on the understanding of their vital role in the regulation of cartilage developmental processes^13^. The use of growth factors has not only enhanced proliferation and differentiation in culture; but has also prompted redifferentiation of chondrocytes upon transfer into a 3D environment and increased both the biochemical and biomechanical properties of formed cartilage with improved migration and integration to the adjacent tissue upon implantation^14–16^. It has also made it possible to promote the expansion of chondrocytes obtained from elderly patients with osteoarthritis^17,18^. The use of growth factors has allowed for the formulation of low percentage autologous serum or even serum-free media, hence solving the controversy over standardization and use of xenogeneic serum in culture^19^.

However, this extensive ex vivo manipulation of chondrocytes using exogenous growth factors raises the issue of their clinical biosafety. Biosafety assessment of ex vivo manipulated cells intended for transplantation is a part of the requirements by many regulatory bodies worldwide, such as the Food and drug administration (FDA) and the European Medicines Agency (EMA)^20^. These agencies consider manipulated cells as “therapeutic products” that need to be strictly regulated and produced according to the good manufacturing practice and under quality control^21,22^. While cell products are usually tested for identity, viability, sterility, and endotoxin level; there are still no standardised comprehensive preclinical protocols to check for genomic instability, small molecular aberrations, or tumorigenic transformation potential^20,23^.

Although in vitro expanded chondrocytes have been used in ACI for many years in thousands of patients, there has been no apparent evidence of malignant transformation^24^. However, concerns about the risk of tumour formation remain. In mature cartilage, chondrocytes cease proliferation, and strictly function in maintaining the homeostasis of the cartilage tissue^25^. Hence, rapid induction of proliferation by growth factors over the normal rate in an artificial environment deficient in “in vivo” regulatory mechanisms may favour DNA damage accumulation, uncontrolled proliferation, and genomic instability; raising the risk of tumorigenic transformation^22^. Moreover, the involvement of growth factors in the initiation and promotion of tumorigenesis has been well documented in a range of human cancers^26,27^. Thus, it is vital that the risk of tumorigenic transformation due to growth factors’ use in vitro be examined. With increasing reports on the incidence of cytogenetic aberrations during cell ex vivo manipulation^28^, only a few attempts were made to investigate genetic alterations and tumorigenicity in chondrocytes induced by growth factors^29^. Therefore, in the current study, we investigated the biosafety of in vitro expanded human nasal septal chondrocytes (hNSCs) in growth factor cocktail supplemented culture medium. Nasal septal chondrocytes have been presented as a promising alternative source to create hyaline-like cartilage tissues^30,31^. We evaluated the growth factor cocktail for its potential to induce DNA damage, *TP53* mutation, aneuploidy, cell cycle and tumour suppressor genes’ expression (p53, p21 and pRB) in hNSCs culture-expanded for six passages. We have also examined the possibility of any tumorigenic transformation of the growth factor cocktail-manipulated hNSCs in vivo in a nude mouse model. This growth factor cocktail composed of transforming growth factor β2 (TGF β2), basic fibroblast growth factor (b-FGF), insulin-like growth factor 1 (IGF-1), insulin-transferrin-selenium (ITS), and platelet-derived growth factor (PD-GF).

## Results

### Effect of growth factors (GF) on hNSC morphology

**Figure 1:**
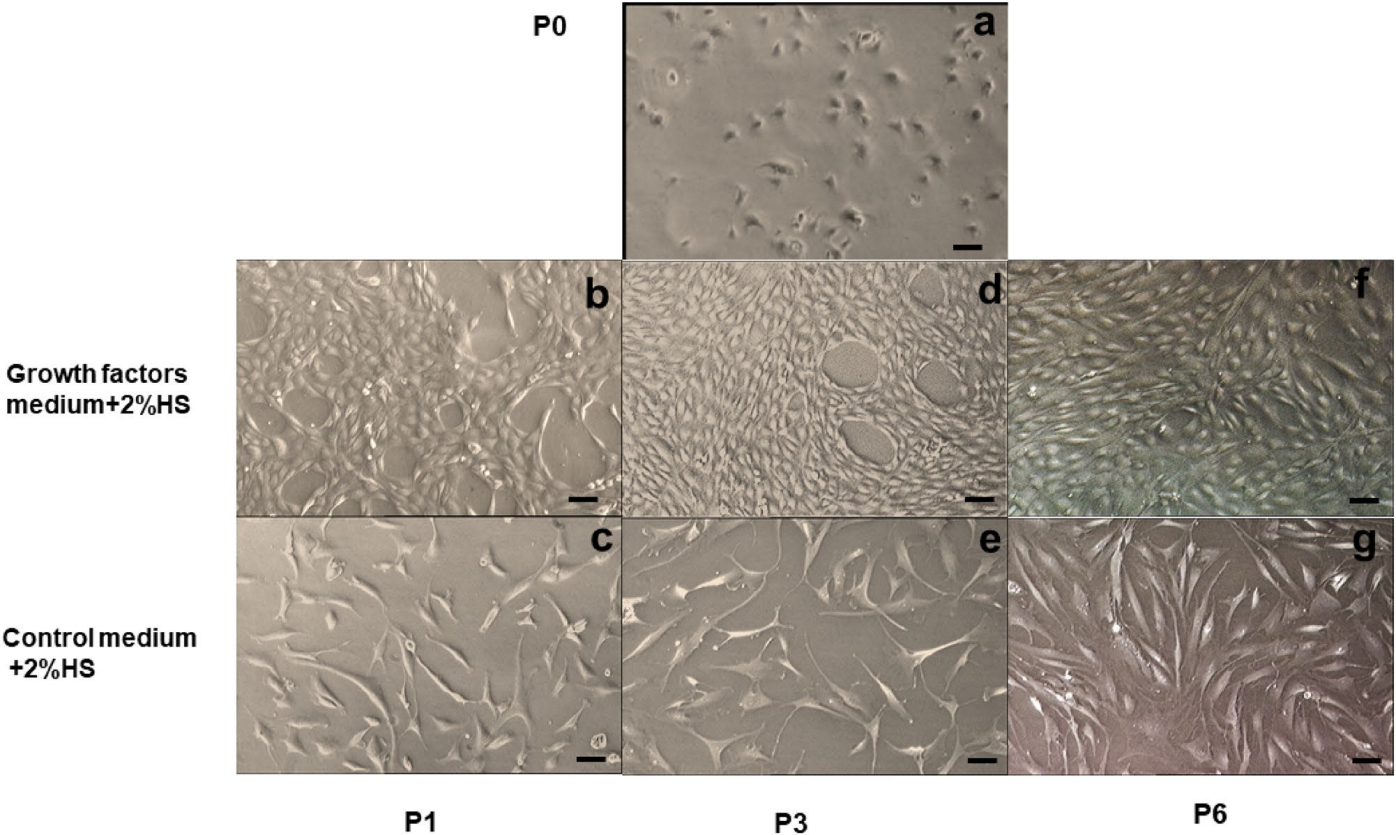
Photomicrographs show human septal chondrocytes morphology (hNSCs). (**a**) GF + 2%HS treated hNSCs at P0 day2; (**b**) GF + 2%HS treated hNSCs at P1 day2; (**c**) Control medium 2%HS at P1 day2; (**d**) GF + 2%HS treated hNSCs at P3 day3; (**e**) Control medium 2%HS at P3 day3; (**f**) GF + 2%HS treated hNSCs at P6 day3, showed mainly polygonal shape chondrocytes; (**g**) Control medium 2%HS at P6 day3. (GF—growth factors; HS—human serum). Scale bar = 10 µm in all images (**a**–**g**) N = 6.

Morphology of chondrocytes is an important indicator of the differentiated status in culture. At initial culture (P0), hNSCs appeared in a polygonal shape (Fig. 1a). At P1, hNSCs of the control group (2%HS) started to assume fibroblast-like morphology (Fig. 1c) while GF-cultured hNSCs maintained the polygonal morphology (Fig. 1b). At P3, hNSCs of the control group appeared larger, more flattened, and with long cytoplasmic extensions with heterogeneous morphology; in comparison with GF-cultured hNSCs that sustained the polygonal morphology with slight elongation (Fig. 1d,e). With passaging, the proportion of fibroblast-like morphology increased in GF-cultured hNSCs;however, they were still smaller with no cytoplasmic extensions observed up to P6 compared with the control group (Fig. 1f,g).

### Growth factors effect on hNSC viability

**Figure 2:**
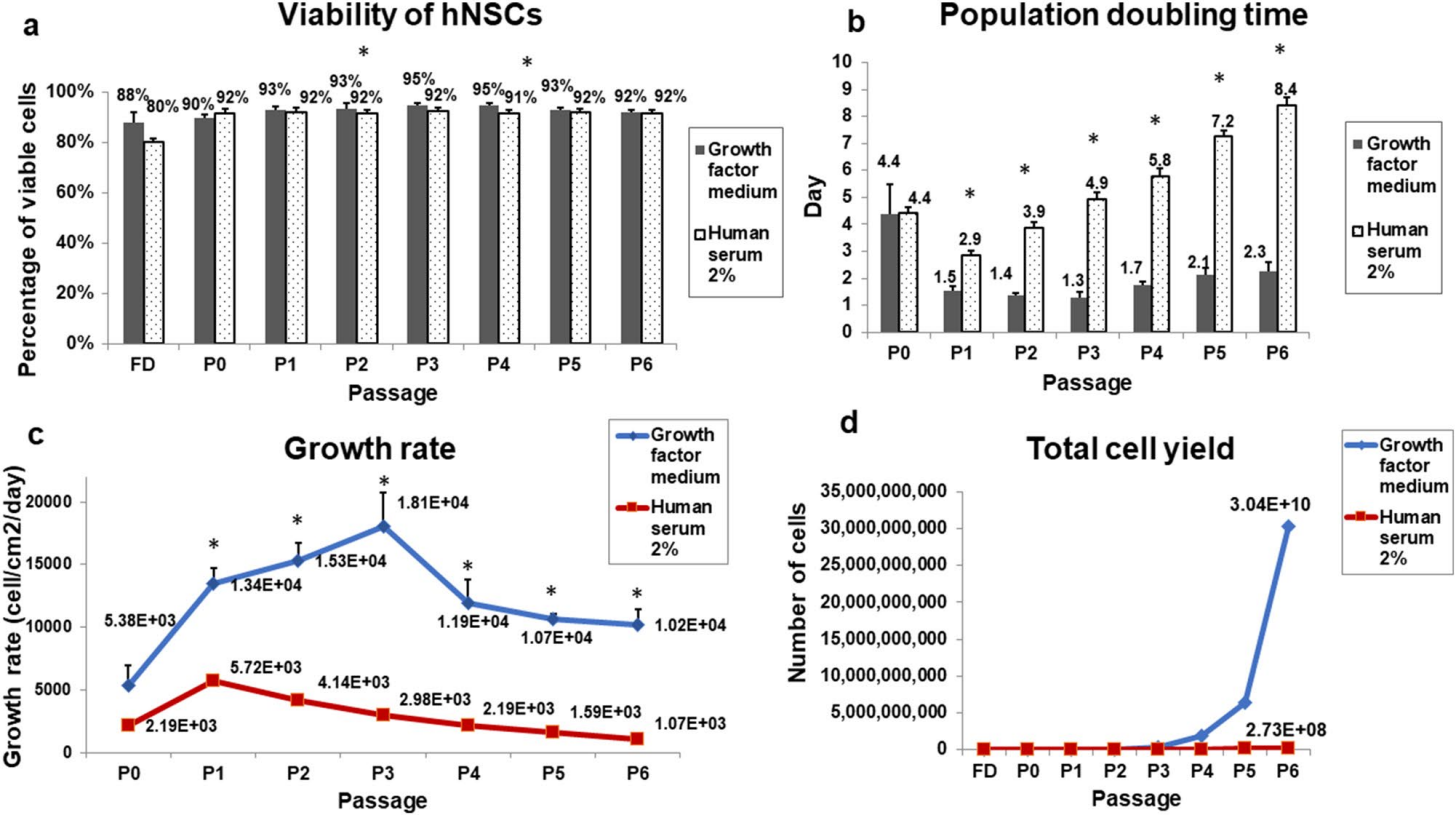
Viability, population doubling time, growth rate, and total cell yield of cultured human nasal septal chondrocytes (hNSCs). (**a**) The mean percentage of viable GF-treated hNSCs based on the trypan blue exclusion test. (**b**) Population doubling time was significantly lower in GF-treated hNSCs compared with the control medium of 2%HS starting from P1 to P6. (**c**) The growth rate (cells/cm^2^/day) was significantly higher in GF-treated hNSCs compared with the control medium of 2%HS starting from P1 to P6. (**d**) Total cumulative cell yield of GF-treated hNSCs and the control medium 2%HS were 3.04 × 10^10^ vs. 2.73 × 10^8^ cells respectively. P—passage number. GF—Growth Factors. (*) represents a statistically significant difference when p < 0.05 as analysed by one-way ANOVA. Values are presented as mean ± SEM, (n = 6).

A simple trypan blue exclusion test was performed at each passage to investigate the effect of GFs cocktail on cultured hNSCs viability. The average proportion of live cells at P0 was about 90 ± 0.01% (Fig. 2a). The highest viability score was observed at P3 and P4 where viability was about 95 ± 0.02 and 95 ± 0.03% respectively. Viability then slightly decreased at P5 and P6 to 93 ± 0.03 and 92 ± 0.02% respectively. Viability was significantly higher in GF-cultured hNSCs group at P2 and P4 (p < 0.05; p = 0.04 and 0.01 respectively).

### Effect of growth factors on hNSC proliferation

The population doubling time (PDT) of GF-cultured hNSCs was significantly lower than the control group (2%HS) from P1 to P6 (p < 0.05; average of 2.2 days in GFs cocktail vs. 5.4 days in control) (Fig. 2b). The lowest PDT in GF-cultured hNSCs was observed at P3; that was 1.28 ± 0.2 days. The highest PDT was at P6: 2.27 ± 0.3 days in the GF-cultured hNSCs, and 8.4 ± 0.3 days in control (p = 0.00). Similarly, the growth rate of GF-cultured hNSCs was significantly higher than the control group throughout the passages starting from P1 onwards (p < 0.05; with an average of 11,826 cells/cm^2^/day in GFs cocktail medium vs. 2840 cells/cm^2^/day in control), (Fig. 2c). The highest proliferative activity was at P3 (18,052.3 ± 2695.7 cells/cm^2^/day) in GF-cultured hNSCs. GF-cultured hNSCs growth rate reached the lowest at P6 (10,165.64 ± 1247.3607cells/cm^2^/day). However, this decline was not statistically significant compared with the previous passages (p > 0.05).

The cumulative of cell doublings from the initial culture to P6 was 16.6 doublings in GF-cultured hNSCs as opposed to 9.8 in the control group. The number of the extracted chondrocytes from the septal tissue ranged between 300–600 × 10^3^ cells. By P6 the total cumulative cell yield was significantly different between the two groups mounting to about 3.04 × 10^10^ in GF-cultured hNSCs in comparison with 2.7 × 10^8^ hNSCs in control medium (Fig. 2d).

### Effect of growth factors on DNA damage by comet assay

**Figure 3:**
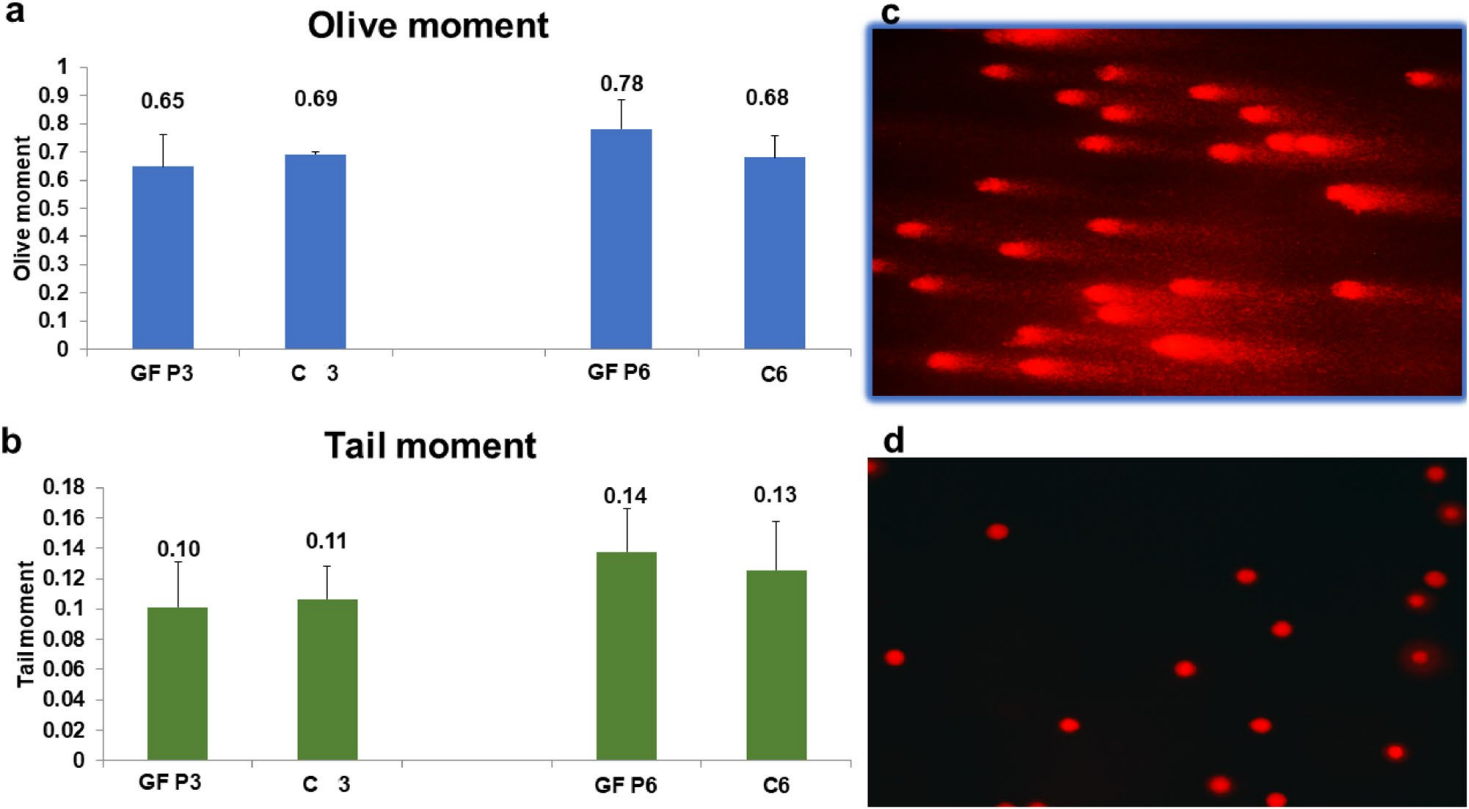
Genotoxicity testing of Growth factor cocktail medium by comet assay. (**a**,**b**) Analysis of DNA damage by comet assay measuring tail moment which is the percentage of DNA in the tail and the mean interval of migration in the tail. Olive moment is the outcome of the proportion of total DNA in the tail and the distance between the centres of head mass and tail area. No significant difference was detected between all groups, p > 0.05, as analysed by two-tailed unpaired Student’s t-test. (**d**) A representative picture of DNA of hNSCs cultured in GFs with an insignificant level of DNA damage. (**c**) A representative picture of damaged DNA of chondrocytes treated with 100 μmol/L hydrogen peroxide. GF—Growth Factors Cultured Chondrocytes. P—passage number; C—Control. Measurement was presented as mean ± SEM, (n = 6).

Comet assay was carried out in this experiment to assess the possibility of GFs becoming genotoxic to hNSC by examining the degree of DNA damage at P3 and P6 of culture (Fig. 3a,b). Tail moments scores of 0.10 ± 0.03 in GF-treated hNSCs vs. 0.11 ± 0.02 in control hNSCs at P3; and 0.14 ± 0.11 GF-treated hNSCs vs. 0.13 ± 0.08 control hNSCs at P6; demonstrating an insignificant level of DNA damage (p = 0.913, 0.802; for P3 and P6 respectively) by means of two-tailed unpaired Student’s t-test. Additionally, no significant difference between GFs samples at P3 compared with P6; (p = 0.398) as determined by two-tailed unpaired Student’s t-test. Likewise, no significant difference in DNA damage between GF-treated hNSCs and control in Olive moments (p = 0.805, 0.557). Figure 3c,d shows representative images of nucleoids of hNSCs treated with H_2_O_2_ as opposed to GF-treated hNSCs, respectively.

### Effect on hNSC cell cycle and DNA ploidy

**Figure 4:**
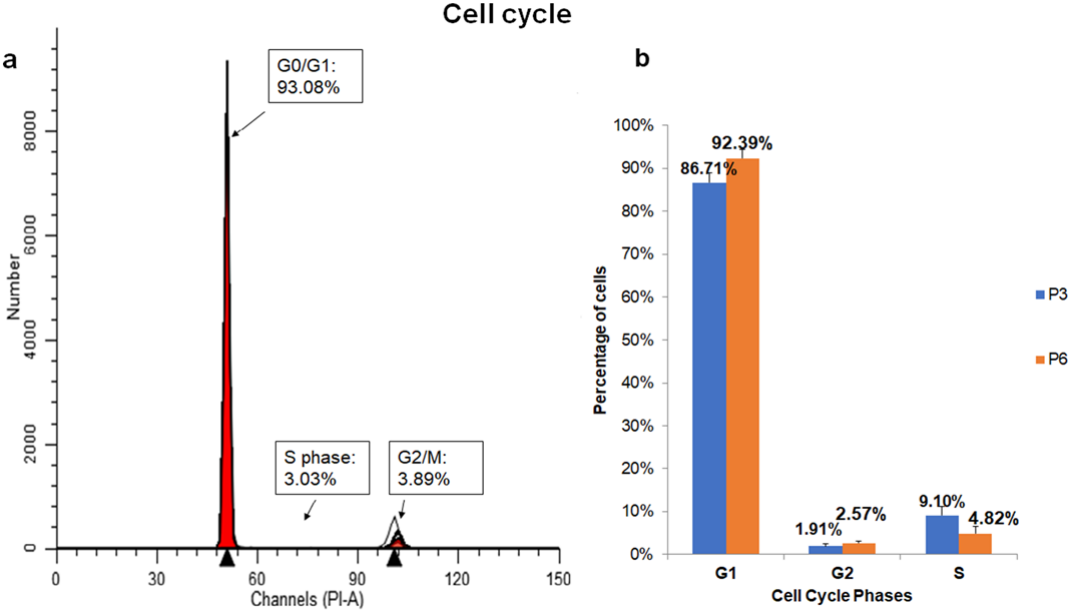
Cell cycle distribution analysis of human nasal septal chondrocytes (hNSCs) cultured in growth factor cocktail medium by flow cytometry. (**a**) A representative single-variable histograms of DNA composition with the percentage of cells at G0-G1, S, and G2-M (at passage 6) shown. The x-axis, the fluorescence of the dye attached to DNA, indicating DNA content and the y-axis indicates number of cells to DNA content. (**b**) Bar graphs representing hNSCs of P6 percentage at the cell cycle phases G0-G1, S, and G2-M at P3 and P6. The percentage of cells was the highest at G0-G1. No statistically significant difference was observed as determined by two tailed unpaired Student’s t-test. Data were presented as mean ± SEM, p > 0.05, n = 6.

The cell cycle was analysed by FACS analysis after GF-treated hNSCs DNA was stained with propidium iodide in a stoichiometric manner, as shown in the bar graphs and DNA single variable histograms (Fig. 4). Most of the cells at P3 and P6 were at the G0/G1 phase (86.71% and 92.39% respectively). The rest of the cells distributed between the S phase (9.10% of P3 cells and 4.82% of P6 cells); and the G2/M phase (1.91% of P3 cells and 2.57% of P6 cells). Interestingly, there was no statistically significant difference between cells at P3 or P6 at each phase of the cell cycle as determined by two tailed unpaired Student’s t-test (p = 0.089, 0.143, 0.470 for G0/G1, S, G2/M phases respectively). No aneuploidy peaks or abnormal cell populations were detected, and all cells were diploids (ploidy: 2 ± 0.01) with a DNA index of 1 ± 0.008 in comparison with the reference diploid cells (PBMCs).

### Effect of growth factors on hNSC tumour suppressor genes expression levels

**Figure 5:**
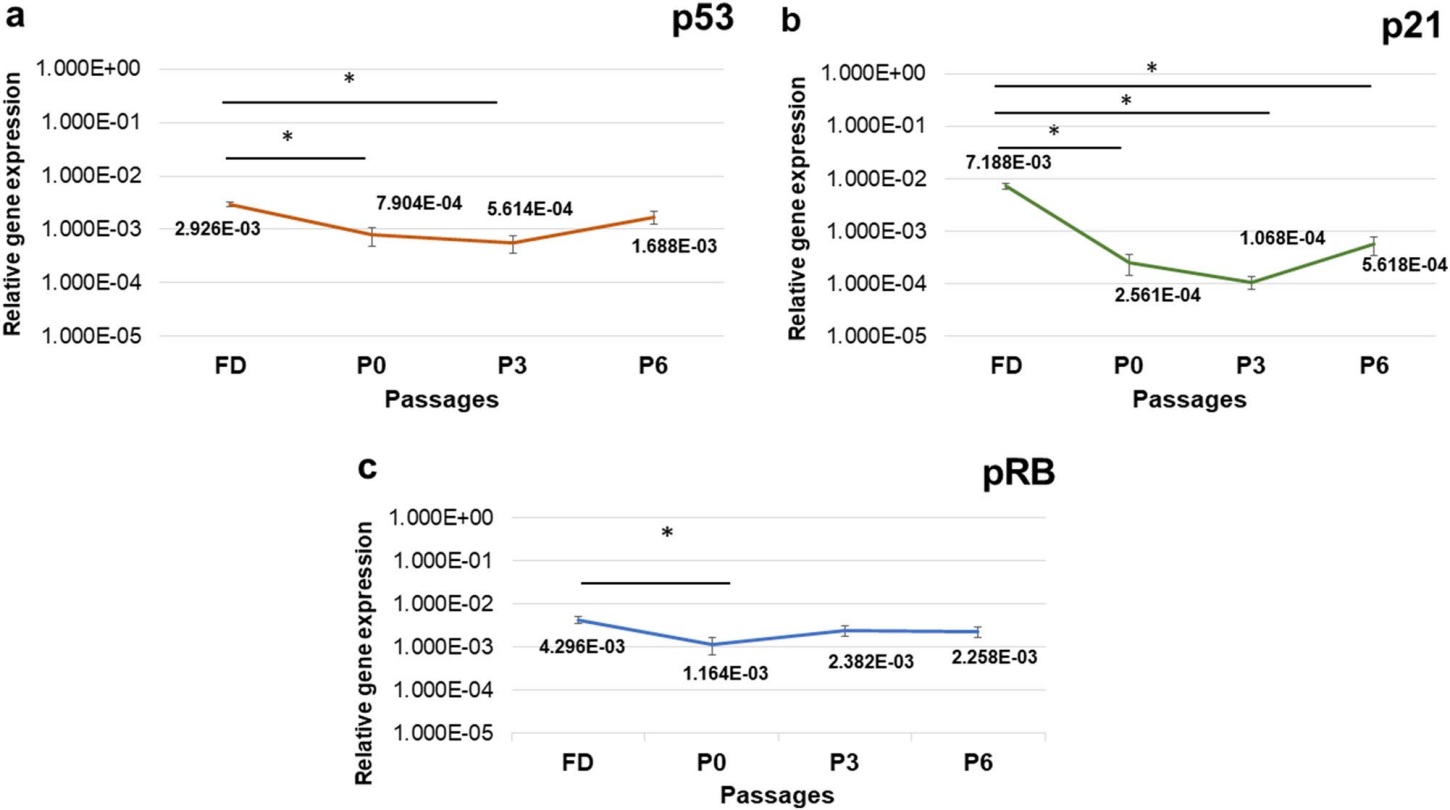
mRNA expression levels of tumour suppressor genes p53, p21, and pRB of human nasal septal chondrocytes cultured in growth factor cocktail medium (hNSCs) in comparison with native cartilage expression (FD). p53, p21, and pRB expression were tested at FD (uncultured chondrocytes, freshly analysed after digestion), P0, P3, and P6 using RT-qPCR. Data were normalized to GAPDH and presented as mean ± SEM. (**a**) p53 showed a significant decrease in expression at P0 and P3 (p = 0.004, 0.002 respectively). (**b**) p21 showed statistically significant downregulation at all passages in comparison with FD (p = 0.00). (**c**) pRB showed a significant decrease in expression at P0 only (p = 0.037). FD- fresh digest, uncultured freshly analysed after digestion. P—passage number. (*) represents a statistically significant difference when p < 0.05 as tested by one-way ANOVA test, (n = 4).

mRNA expression analysis of p53, pRB, and p21 of GF-cultured hNSCs, revealed a trend of decreased expression levels in comparison with their expression in the uncultured freshly digested cells (FD) that represent expression in the native tissue. p53 showed a statistically significant decrease in expression levels at P0 and P3 compared with FD (p = 0.004, 0.002, respectively; Fig. 5a). This was followed by a gradual increase at P3 through P6. p21 showed statistically significant downregulation at all passages in comparison with FD (p = 0.00; Fig. 5b). pRB showed a significant decrease in expression at P0 only compared with FD (p = 0.037; Fig. 5c). Confirming the specificity of the qPCR reaction, distinct single peaks were detected in melting curve for each amplified product. Gel electrophoresis as well revealed a single specific band for each amplified product and of the right size.

### TP53 mutation detection

**Figure 6:**
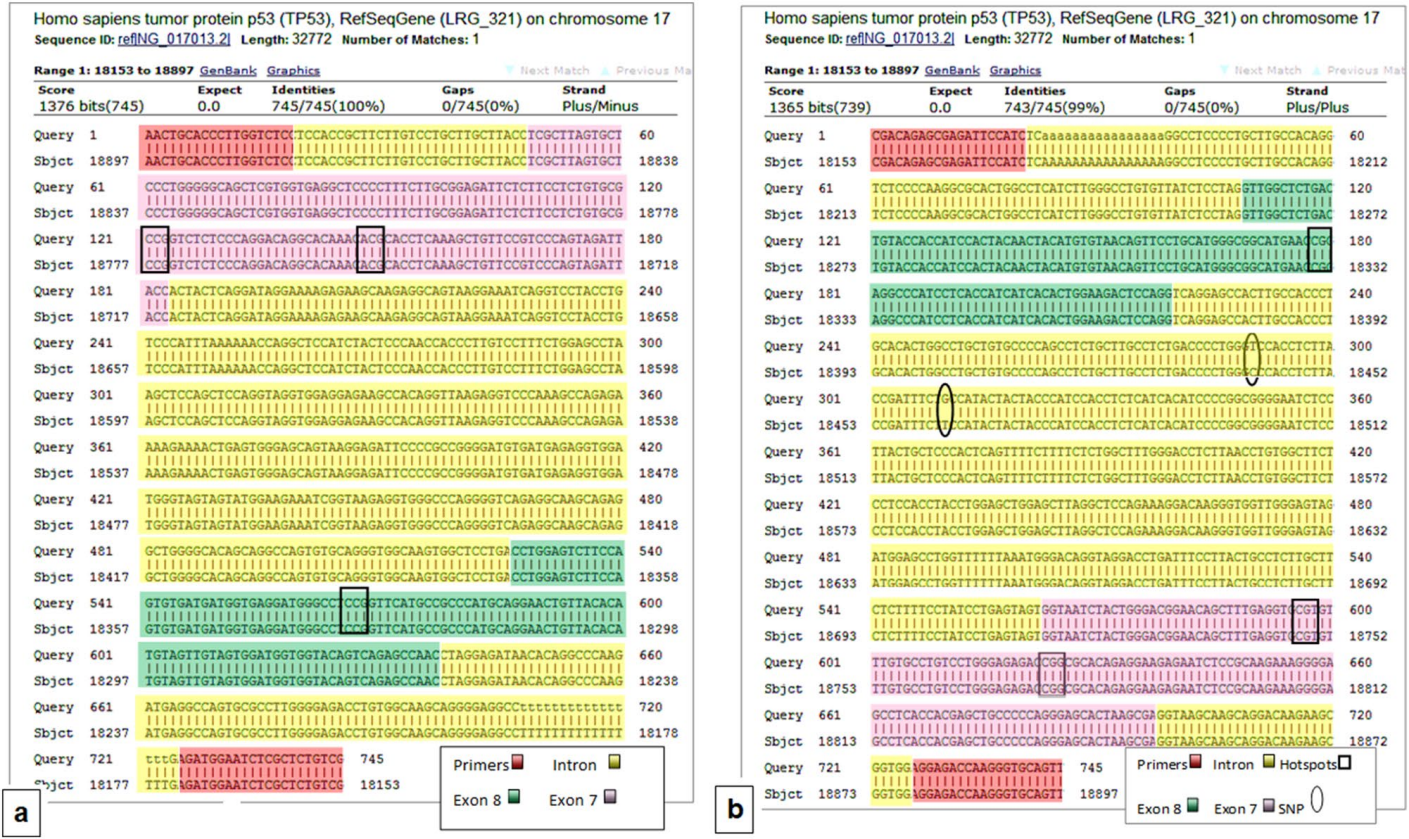
Illustration of the alignment of DNA nucleotide sequence of chondrocytes cultured in growth factor medium with wild-type *TP53* using Basic Local Alignment Search Tool (BLAST) software. (**a**) Alignment with wild-type *TP53* (*forward strand*). Locations of primers, exons, and introns are shown. Hotspot amino acids Arg248 is located on exon7; Arg 273 and Arg 282 are located on exon 8. 50% of subjects had 100% match wild-type *TP53*. (**b**) Alignment with wild-type *TP53* (*reverse strand*). Single point polymorphisms (SNPs) in the intron 7 were at two positions 18442 C substituted with T and 18462 where T substituted with G as shown above. This represents 50% of the subjects. Gene reference: NG_017013.2. N = 6.

Sequences of GF-cultured hNSCs *TP53* (n = 6) were compared with wild- type *TP53* tumour suppressor gene for mutation detection using BLAST. None has exhibited any kind of mutations in the coding gene and the hotspot areas. Three samples exhibited single nucleotide polymorphisms (SNPs) in intron 7- the area between exon 7 and exon 8 (Fig. 6a,b). The polymorphisms were at two positions: 18442 C was substituted with T and 18462 where T was substituted with G (gene reference: NG_017013.2).

### Tumorigenic transformation in nude mice model

**Figure 7:**
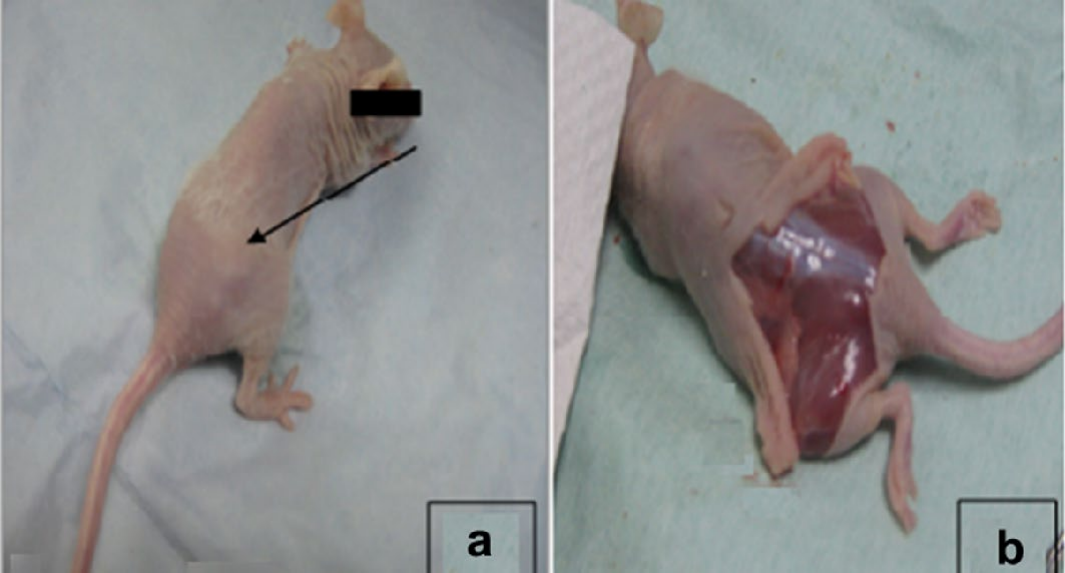
Tumorigenicity test of nasal septal chondrocytes cultured in GFs until P6 implanted into nude mice model for 6 months. (**a**) Palpation and macroscopic examination of any changes in skin appearance or lump formation, at the implantation area of the dorsal subcutaneous part of the nude mouse. No abnormal growth of tissue was observed. (**b**) Examination of the area of implantation after the skin was removed, there was no tumour produced, no evidence of metastasis, superficial infection, or fistula formation.

After 6 months of observation, the fibrin constructs of P6 GF-cultured hNSCs did not form tumours in the immunodeficient mice. None of the mice presented weight loss. Organs appeared macroscopically normal. No evidence of tumorigenic transformation, metastasis, superficial infection, or fistula formation (Fig. 7).

## Discussion and conclusion

The limited availability of chondrocytes is a major challenge for cartilage tissue engineering. Monolayer expansion is necessary to cultivate chondrocytes. In vitro expanded chondrocytes dedifferentiate and undergo senescence and their proliferative and chondrogenic capabilities decrease^8^. Therefore, a mixture of chondro-inducive and mitogenic growth factors is often used in clinical settings to improve monolayer culture conditions^31,32^.

While some previous studies demonstrated genetic instability had taken place in a long term ex vivo culture (with no growth factors) due to replicative stress^28,29^, others suggested long term ex vivo expansion to be safe^30,33^. Consequently, we were curious to investigate the effect of the stress growth factors might induce on human chondrocytes in long term culture- especially that studies on the biosafety of in vitro manipulated chondrocytes with growth factors are still limited. Hence, in the present study, we assessed the possibility of a cocktail of growth factors, i.e. transforming growth factor β2 (TGF β2), basic fibroblast growth factor (b-FGF), insulin-like growth factor 1 (IGF-1), insulin-transferrin-selenium (ITS), and platelet-derived growth factor (PD-GF) to induce genetic instability and tumorigenicity in human nasal septal chondrocytes (hNSCs) in vitro and in vivo.

Our data show that growth factor cocktail stimulated an intense proliferative drive on hNSCs. It shortened population doubling time, increased growth rate, and displayed a low risk of cytotoxicity (cell viability was more than 90% even at P6). These findings are in concordance with previous studies, whereby growth rate and population doublings were significantly enhanced with the use of growth factors^32,34^. Conversely, in the current experiment, 2% human serum supplementation alone has poorly supported hNSCs growth. Morphologically, the growth factor treated hNSCs’ polygonal shape was maintained until P3. However, the fibroblast-like feature has increased at higher passages. Although we did not test for senescence markers, our results showed no morphological indications of senescence, and hNSCs were still actively proliferating up to P6 (16.6 population doublings). Generally, morphological characteristics of senescent cells include increased cell size, flattening, increased cytoplasmic extensions, heterogeneous morphology, and halted proliferation^35^. However, none of these features were noted in this study. Without growth factors, senescence features in chondrocytes occurred as early as eight population doublings^36^. Hence, it is reasonable to deduce that the growth factor cocktail could shorten the culture time by boosting cell growth rate with a low risk of senescence and cytotoxicity.

Adding to this, in this study, the use of serum was minimised to only 2% of human serum. Serum—either of human or animal source-has been widely used to promote ex vivo attachment and proliferation of cells due to the composition of abundant growth factors^37^. Our data show that this reduction has not affected the proliferation competency of chondrocytes. Rather, this reduction was compensated with the distinct combination of growth factors that is contained in the cocktail. Thus, the growth factor cocktail could help to minimise the use of autologous serum in culture that is usually 5–10%^31,38^; or 10% fetal bovine serum^39^ in the current practice. Consequently, this may provide a solution to the problem of the limited autologous serum supply and reduce the effect of serum variation on the reproducibility and the quality control of the generated tissues.

The increase in proliferation ability prompted us to investigate the growth factor cocktail’s potential to cause genotoxicity by inducing DNA damage and chromosomal aberrations (aneuploidy) in culture. We hypothesised that the replicative stress induced by the growth factor cocktail—in a microenvironment that lacks in vivo regulatory mechanisms—may disrupt cell cycle regulation supporting DNA damage accumulation and genetic instability^22^. This biosafety element is vital to verify since sporadic tumours were found to arise from the accumulation of DNA lesions^40^; and early stages of human tumours have a high occurrence of DNA damage and aneuploidy^40,41^. If on one hand DNA damage accumulation can activate cell DNA damage response (DDR) inducing repair or cell cycle arrest (apoptosis and senescence); on the other hand, it can prompt genetic instability amplifying the potential of transformation^42,43^. For that reason, we employed comet assay for its ability to detect DNA double strand breaks, single strand breaks, alkali labile sites, oxidative DNA base damage, DNA–DNA, and DNA–protein repair^44^. Our comet assay results showed that the growth factor cocktail did not cause increased DNA damage even for a considerably long-term exposure up to P6. We further employed flow cytometry to evaluate DNA content to detect any cell clones with abnormal chromosomal content (aneuploidy)^45^. Our results showed no aneuploidy peaks or abnormal cell populations consistent with Kamil’s et al. observation^46^. We also observed a similar synthesis phase fraction (SPF) to previous studies^46,47^ and normal cell cycle profile with most cells at G1.

Analysis of key DDR tumour suppressor genes p53, p21, and pRB expression uncovered a significant trend of decreased expression in comparison with the native tissue. This was followed by a gradual increase towards P6 to normal levels (p53 and pRB only). Of all cell cycle regulators, the *p53* transcription factor stands out as a key tumour suppressor and a vital controller of different signalling pathways involved in tumorigenesis^43^. As a main DDR protein, p53 triggers cell cycle arrest to allow for DNA repair, senescence and apoptosis^48^. Therefore, increased expression of p53 above the basal level is indicative of increased various types of stresses. When p53 is activated, it interacts with p21 (cyclin-dependent kinase inhibitor) to inhibit cdk2 hence arresting cell cycle at G1 and G2/M^48^. Inhibiting cdk2 causes dephosphorylation of the tumour suppressor pRB^48,49^. pRB protein interacts with E2F transcription factor when dephosphorylated forming a complex, thus halting cell cycle progression at G1 as E2F is essential for transcription of genes controlling the cell cycle progress^48,49^. p53 and p21 also can suppress cell cycle through p53–p21–DREAM–E2F/CHR pathway (p53–DREAM pathway)^50^. Alternatively, the dysfunction of these genes may allow for uncontrolled cell proliferation and neoplastic transformations^43^. In the present study, the observed decreased expression of these vital genes could be explained by p53 role in the regulation of plasticity and differentiation during regeneration^51^. It has been proposed that the downregulation of p53 is necessary to permit postmitotic differentiated cells to return to the cell cycle^52^. p53 inhibition was suggested to be essential for the formation of proliferative progenitor cells in the regenerating tissue recapitulating the early developmental stages of tissues^52^. Consistent with previous studies, we noted the downregulation of p53 is followed by the downregulation of its other effector genes, including p21 and pRB^48,49^. Hashimoto and coresearchers proposed downregulation of p53 expression can prevent chondrocytes from undergoing apoptosis or senescence^53^. Interestingly, Kim et al. correlated downregulation of p21 with the enhanced proliferative capacity, and chondrogenic phenotype of chondrocytes expanded in vitro^54^. They further showed p21 was upregulated just before cell cycle arrest. This may explain why p21 in the current study maintained low levels, even up to P6, which correspond with our growth rate data. Taken together and supported with our earlier data of low DNA damage and absence of aneuploidy findings, the cocktail medium induced a transient downregulation of tumour suppressor genes and did not distort hNSCs cell cycle regulation nor induce uncontrolled proliferation.

For further confirmation, we studied the possibility of mutation emergence in *TP53* at the hotspot areas of exons 7 and 8. These exons contain three amino acid hotspots that were found to have a high occurrence of mutations in most cancers^55,56^. These are Arg 248 located on nucleotides 742 and 743 of Exon 7, Arg 273 located on nucleotides 817 and 818; and Arg 282 located on nucleotides 844. Missense mutations in the *TP53* gene are tremendously prevalent in human cancers. Mutated *TP53* gains oncogenic functions impairing cell DDR, thus promoting uncontrollable cell growth and tumorigenicity^57^. In the present study, overall, our sequencing analysis revealed that there was “no mutations” in the coding gene and the hotspot areas of all tested samples. Three samples (50% of subjects) exhibited single nucleotide polymorphisms (SNPs) in the intron 7- the area between the exon 7 and exon 8. The polymorphisms were at two positions: 18442 where C was substituted with T, and 18462 where T was substituted with G. These SNPs have been previously reported as minor alleles related to ethnicity-based variations^58^.

Furthermore, we examined the possibility of tumorigenic transformation in vivo. Fibrin-constructs made from hNSCs cultured in growth factor cocktail till P6 were transplanted subcutaneously at the dorsal part of immunodeficient mice for 6 months. Tumorigenicity testing in immunodeficient mice was approved in clinical trials^31^. Theoretically, these cells have a greater risk of tumorigenic transformation due to the extensive ex vivo manipulation^22^. Though hNSCs of P6 failed to form neocartilage as a result of dedifferentiation at this late passage; we found no evidence of tumour development or metastasis subsequent to 6 months of inspection.

It is important to note that this study has a potential limitation concerning the use of varying controls between the different experiments. This was due to the limited available cells from the source material (patient’s cartilage sample) and the insufficient number of cells generated from the control group. This consequently has constrained the use of a consistent control group throughout the study. Yet, the selection of the respective control groups was carefully made to meet the objective of each experiment.

Collectively, our findings come in agreement with previous reports^29,46,59^. Our data revealed no evidence of genotoxicity, cytotoxicity, disturbance of DDR tumour suppressor gene expression (p53, p21 and pRB), aneuploidy, or *TP53* mutation. Normal cell cycle profile was observed with no uncontrolled proliferation or tumour formation in vivo for as long as 6 months. This study is among the few studies which addressed the modifications that growth factors might exert at the cellular, genetic and small molecular levels and their effect on the biosafety of the cultured cells.

Lately, there has been a growing concern regarding the biosafety of the ex vivo expanded cells. Contradictory reports on the incidence of cytogenetic alterations at progressive culture times^28–30,33,60^ may have arisen from the fact that the employed techniques greatly differed in their sensitivity. Moreover, the vast majority of current techniques—including the techniques used in this study—are unable to sense anomalies arise in below 10% of the examined cells^22^. There is also a possibility of amplifying anomalies that are already present in cell donor^61^. Therefore, it is essential to develop a universally recognised, case-by-case strategy to check for genetic instability and tumorigenic transformation of therapeutic cell products before infusion to patients^23^. Lack of consensus is an obstacle to the advancement of cell therapy approach, particularly that the risk of tumorigenic transformation and genetic disruption due to ex vivo manipulation of therapeutic cells remains. At this point of time and based on our data, our study provides support to the biosafety profile of growth factor cocktail-expanded human septal chondrocytes (hNSCs) for six passages and 16.6 population doublings showing a low risk to induce genotoxicity or neoplastic transformation.

## Materials and methods

### Isolation and culture of human nasal septal chondrocytes (hNSCs)

Redundant human nasal septum tissues were collected from six patients, who underwent an elective septoplasty. Informed consent was obtained from all donors before the surgery. Donors aged between 24 and 52 years. Collection of discarded human nasal septum tissues and performance of experiments concerning human specimens were with the approval from the Medical Research Ethics Committee Universiti Kebangsaan Malaysia (UKM 1.5.3.5/244/UKM-DLP-2011-070) and in accordance with the guidelines and regulations stipulated in the Declaration of Helsinki. Nasal septum specimens were cleaned from perichondrium, mechanically minced, and digested in 0.6% Collagenase Type II buffer at 37 °C for 5–6 h in a shaker. After digestion, isolated hNSCs were quantified and assessed for viability using trypan blue exclusion test.

### Cell expansion assessment

Isolated hNSCs were seeded as the primary culture (P0) with a density of 10,000 cells/cm^2^ in 6-well tissue culture plates (Nunc, Denmark) in an equal volume mix of Ham’s F12 and Dulbecco’s Modified Eagle Medium (F12: DMEM), supplemented with 1% antibiotic–antimycotic, 1% Glutamax and 1% vitamin C (Invitrogen). The experimental medium was supplemented with 2% human AB serum (Sigma-Aldrich) with growth factor cocktail (GFs) of TGF β2, b-FGF, IGF-1, ITS and PD-GF (Gibco, Grand Island, NY, USA). While the control group medium was supplemented with 2% human serum only. Cell cultures were incubated at 37 °C with 5% carbon dioxide. Medium was changed every three days. Once 80–90% confluent, cells were sub-cultured using TrypLE Select (Gibco) and serially expanded until passage six (P6). Cell count and viability were assessed by trypan blue exclusion test. Population doubling time (PDT), and growth rate (GR) were determined according to the following equations:

1 $${\text{PDT }}\left( {{\text{days}}} \right) = \, \left[ {{\text{ N}}_{{\text{c}}} /{\text{N}}_{{\text{d}}} } \right]$$

(N_c_ : days in culture; N_d_ : doubling number in each passage).

N_d_ was calculated according to this formula:

$${\text{N}}_{{\text{d}}} = {\text{Log }}\left[ {{\text{N}}_{{\text{e}}} /{\text{N}}_{{\text{s}}} } \right]/{\text{Log2}}$$

2 $${\text{Growth rate }}\left( {{\text{cells}}/{\text{days}}/{\text{cm}}^{{2}} } \right) = \left[ {\left( {{\text{N}}_{{\text{e}}} /{\text{N}}_{{\text{s}}} } \right)/{\text{d}}/{\text{surface area}}} \right]$$

(N_e_: cell quantity by the ending of culture; N_s_: cell quantity by the beginning of culture, d: duration of culture in days).

### Genotoxicity assessment by comet assay

hNSCs at the third passage (P3) and the sixth passage 6 (P6) were examined for DNA damage using the comet assay (n = 6). Briefly, about 20,000 cells in 30 µl of chilled PBS were mixed with 80 µl of 1% low melting point agarose. The mixture was pipetted to fully frosted slides precoated with a layer of 1% normal melting point agarose. The mixture was spread by using coverslips, and slides were incubated at 4 °C for 20 min for the gel to harden. Then, coverslips were removed, and slides were incubated in lysis solution (2.5 M NaCl, 0.1 M EDTA, 0.01 M Tris and 1% Triton X-100 and 1% DMSO) at 4 °C overnight to enhance sensitivity. Slides were incubated for 20 min in electrophoresis buffer (1 mM EDTA, 300 mM NaOH, pH 13) to allow DNA unwinding before being electrophoresed at 25 V and 300 mA for 20 min. Slides were then washed with neutralisation buffer (0.4 M Tris buffer, pH 7.5) for 5 min and stained with 5 mg/ml of ethidium bromide. Slides were viewed by a fluorescent microscope with a 590 nm filter. 50 comet images were randomly captured for each slide and analysed for tail moment and Olive tail moment that are automatically acquired using CometScore version 1.5.2.6 software (Tritek Corp., Sumerduck, VA, USA). The tail moment is described as the outcome of the tail length and the proportion of overall DNA in the tail, whereas, Olive moment is the outcome of the proportion of overall DNA in the tail and the interval between thecentres, the head mass and tail area^62^. Chondrocytes treated with 100 μmol/L hydrogen peroxide for 1 h, a treatment identified to result in DNA breaks, were used as a positive control^63^. Untreated hNSCs were used as treatment control. This experiment was carried out in triplicate for each sample.

### Cell cycle and DNA ploidy assessment by flow cytometry

Flow Cytometry test was employed to evaluate DNA contents of GF-cultured hNSCs at P3 and P6 (n = 6) using CycleTEST Plus DNA Reagent Kit (Becton Dickenson, San Jose, CA). Cells were stained with propidium iodide according to the manufacturer’s instructions. A minimum of 5.0 × 10^5^ hNSCs per sample were stained with propidium iodide in suspension and analysed by FACSCanto II Flow Cytometer (BD Bioscience). At least 2 × 10^4^ events were acquired. Results obtained of distribution of cells at G0/G1, S phase, and G2 were analysed by MODFIT-LT software programme version 3.3 (Verity House Software, Topsham, ME, USA; http://www.vsh.com/products/mflt/index.asp). Human peripheral blood mononuclear cells (PBMCs) were used as a control. The ratio of hNSC G0-G1 peak scores to PBMCs G0-G1 peak scores was expressed as a DNA index. A DNA index of 1.0 signifies a normal diploid DNA, whereas values apart from 1.0 designate DNA aneuploidy.

### Cell cycle regulator gene expression analysis by real-time reverse transcriptase quantitative polymerase chain reaction (RT-qPCR)

Total RNA was extracted from fresh digest samples (FD-uncultured) and GF-cultured hNSCs at FD, P0, P2, P3, and P6 upon confluence (n = 4) with Tri-Reagent (Molecular Research Center, Cincinnati, OH, USA) according to the manufacturer’s guidelines. Polyacryl gel carrier (Molecular Research Centre) was used to enhance precipitation of isolated RNA. The resultant pellets were washed with 75% ethanol, air-dried, and resuspended in 20 µl of RNase and DNase-free water. RNA concentration, purity, and quality were estimated using Nanodrop (Thermo Scientific, Waltham, USA). The UV absorbance of the diluted RNA samples was measured at 260 nm and 280 nm as well as the ratio between these two readings. This ratio provides an estimate of the purity of the extracted RNA. In our experiment, only preparations of RNA that have OD ratio values (A_260_/A_280_) of between 1.8 and 2.1 were considered for RT-qPCR. Isolated RNA was kept at − 80 °C. Alterations in tumour suppressor gene p53, human cyclin-dependent kinase inhibitor (p21), and retinoblastoma (pRB) gene expression were investigated by RT-qPCR at P0, P2, P3, and P6. Glyceraldehyde phosphate dehydrogenase (*GAPDH*) was the reference gene. All primers used in this experiment were designed with Primer 3 software and compared with the GenBank database published sequences (https://www.ncbi.nlm.nih.gov/tools/primer-blast/) (sequences in Table 1). Complementary DNA (cDNA) was synthesised by SuperScript III First-Strand Synthesis SuperMix kit for two step RT-qPCR (Invitrogen) according to the manufacturer’s protocol using the following conditions: 25 °C for 10 min, 45 °C for 30 min, 90 °C for 5 min and 37 °C for 20 min. The PCR reaction was performed in Bio-Rad iCycler machine (Bio-Rad Laboratories, USA) using SYBR Green as an indicator with reaction profile of pre-denaturation for 1 cycle for 3 min at 95 °C; PCR amplification for 40 cycles at 95 °C for 10 s, annealing and elongation at 61 °C for 30 s. Melting curve analysis was performed to check for any contaminating or off-target amplification products presented by multiple peaks in the curve. Additionally, resultant RT-qPCR products were run in gel electrophoresis to check for the presence of nonspecific amplification. Relative quantification of all target gene expression was determined by normalisation to glyceraldehyde-3-phosphate dehydrogenase (*GAPDH*) according to the ΔCt analysis (2^ΔΔCt^).

**Table 1:** Primers of genes analysed in RT-qPCR.

Gene	Accession no	Gene description	Primer sequence	PCR product size (bp)
*GAPDH*	NM_002046	Human glyceraldehyde 3-phosphate dehydrogenase	F: 5′-tcc ctg agc tga acg gga ag-3’ R: 5′-gga gga gtg ggt gtc gct gt-3’	217
*P21*	NM_002577.3	Human cyclin-dependent kinase inhibitor 1	F: 5′-gatggcaccagaggtggtta-3’ R: 5′-tcccgaaatattggggaaag-3’	198
*P53*	NM_000546.3	Human tumour suppressor gene	F: 5′-ggaagagaatctccgcaagaa-3’ R: 5′-agctctcggaacatctcgaag-3’	177
*RB*	NM_000321	Human retinoblastoma gene	F: 5′-cagacccagaagccattgaa-3’ R: 5′-ctgggtgctcagacagaagg-3’	115

(1) *GAPDH*: Human Glyceraldehyde 3-phosphate dehydrogenase as a reference gene. (2) *P21*: Human Cyclin-Dependent Kinase Inhibitor 1. (3) *TP53*: Human Tumour Suppressor gene. (4) *PRB*: human retinoblastoma gene. The primer sequences were designed using a wild-type *TP53* sequence provided by the GenBank—the NIH genetic sequence database.

### TP53 mutation detection

DNA was extracted from about 3 × 10^6^ GF-cultured hNSCs at P6 (n = 6) using Wizard Genomic DNA Purification Kit (Promega, Madison, WI, USA) according to the manufacturer’s protocol. Amplification of *TP53* was performed by polymerase chain reaction (PCR). The proofreading Taq Polymerase, Platinum Taq DNA Polymerase High Fidelity (Invitrogen, Carlsbad, CA) was used in this reaction with the reaction profile of 3 min pre-denaturation at 94 °C followed by 35 cycles of denaturation for 30 s at 94 °C, annealing for 30 s at 61 °C, elongation for 1 min at 72 °C, and final elongation at 72 °C for 5 min. The primer sequences were designed using a wild-type *TP53* sequence provided by the GenBank—the NIH genetic sequence database. *TP53* specific forward and reverse primers were 5′-CGACAGAGCAGATTCCATC-3′, and 5′AACTGCACCCTTGGTCTCCT-3′; respectively with a PCR product size of 754 bp. The PCR products were run in electrophoresis on a 1.5% agarose gel and visualised by UV transillumination. The specificity of the PCR was confirmed and *TP53* DNA bands at 745 bp were excised and subsequently purified with PureLink Quick Gel Extraction Kit (Invitrogen). PCR products were cloned into the pTOP TA V2 plasmid vector by the TOP Cloner Blunt kit (Enzynomics, Seoul, Korea) and transformed into chemically competent *Escherichia* *coli* cells (DH5α) according to the manufacturer’s recommendations. Transformed *Ecoli* were plated to agar plates and incubated overnight at 37 °C. Three clones were picked for each sample and inoculated into LB broth for overnight incubation at 37 °C. Clones were confirmed for positivity for *TP53* PCR product by EcoR1 (Invitrogen) restriction enzyme digestion and PCR under the same conditions as mentioned above. Plasmids containing *TP53* PCR products were purified using PureLink Quick Plasmid Miniprep Kit (Invitrogen) and sent for sequencing. *TP53* DNA sequences were compared with wild- type *TP53* for mutation detection employing Basic Local Alignment Search Tool (BLAST) (https://blast.ncbi.nlm.nih.gov/Blast.cgi). Sequencing quality was assessed using Sequencing Analysis Software v5.3 (Applied Biosystems; https://www.thermofisher.com/order/catalog/product/4474950#/4474950).

### Tumorigenicity test of hNSCs in nude mice model

Animal experiments were approved by Universiti Kebangsaan Malaysia (UKM) Animal Ethics Committee (FP/FISIO/2011/CHUA/30-NOVEMBER/411-NOVEMBER-2011-OCTOBER-2013) and conducted according to animal ethics guidelines. To evaluate tumorigenesis in vivo, around 3 × 10^7^ hNSCs at P6 were mixed with fibrin (Tisseel; Baxter), that is derived from human pooled plasma, to form a mass (construct) according to the manufacturer’s recommendation. Six male nude mice weighed 20 ± 2 g and aged around 8 weeks old were used in this experiment. The nude mice were anaesthetised by injecting Zoletil into the rear leg muscles, and the drug was allowed to take effect for 10 min. Each construct was transplanted subcutaneously at the dorsal part of the nude mice (n = 6). Post-operation, the animals were kept in a pathogen free environment in a specialised facility at UKM Animal House, Bangi, Malaysia. Each mouse was kept in a single cage and provided sterile water and food ad libitum. The temperature was maintained at 21 °C and humidity at 50% with 12:12 h light/dark cycle. Animals’ weight was assessed frequently. Tumour development was checked regularly by palpation to detect any abnormal tumour growth and by macroscopic examination of the skin at the site of implantation. After 6 months, the animals were sacrificed by cervical dislocation. The skin at the site of implantation was removed, and the underlining tissue was macroscopically checked for any inflammation, necrosis, discolouration or lump formation. Additionally, animals were dissected, and main organs were checked for masses and macroscopic changes.

### Statistical analysis

IBM SPSS Statistics 21.0 (IBM Corp., Chicago, IL; https://www.ibm.com/my-en/analytics/spss-statistics-software) used to perform data analysis. One-way analysis of variance (ANOVA) with a Tukey post hoc to compare data among the specified groups in each experiment; or two tailed unpaired Student’s t-test as needed. Data were demonstrated as mean ± standard error of mean (SEM). Normality of the data was tested before any statistical analysis using Shapiro Wilk’s test. Statistical significance was set at p < 0.05.

## Abbreviations

hNSCs: Human nasal septal chondrocytes
GF: Growth factor
ACI: Autologous chondrocyte implantation
P: Passage
DDR: DNA damage response

## References

[1] Candela ME, Yasuhara R, Iwamoto M, Enomoto-Iwamoto M (2014). Resident mesenchymal progenitors of articular cartilage. Matrix Biol..

[2] Brittberg M (1994). Treatment of deep cartilage defects in the knee with autologous chondrocyte transplantation. N. Engl. J. Med..

[3] Carey JL (2020). Autologous chondrocyte implantation as treatment for unsalvageable osteochondritis dissecans: 10-to 25-year follow-up. Am. J. Sports Med..

[4] Fossum V, Hansen AK, Wilsgaard T, Knutsen G (2019). Collagen-covered autologous chondrocyte implantation versus autologous matrix-induced chondrogenesis: A randomized trial comparing 2 methods for repair of cartilage defects of the knee. Orthop. J. Sports Med..

[5] Boehm E, Minkus M, Scheibel M (2020). Autologous chondrocyte implantation for treatment of focal articular cartilage defects of the humeral head. J. Should. Elb. Surg..

[6] Wuest SL (2018). Influence of mechanical unloading on articular chondrocyte dedifferentiation. Int. J. Mol. Sci..

[7] Ma B (2013). Gene expression profiling of dedifferentiated human articular chondrocytes in monolayer culture. Osteoarthr. Cartil..

[8] Ashraf S (2016). Regulation of senescence associated signaling mechanisms in chondrocytes for cartilage tissue regeneration. Osteoarthr. Cartil..

[9] Giovannini S, Diaz-Romero J, Aigner T, Mainil-Varlet P, Nesic D (2010). Population doublings and percentage of S100-positive cells as predictors of in vitro chondrogenicity of expanded human articular chondrocytes. J. Cell. Physiol..

[10] Hayes AJ, Hall A, Brown L, Tubo R, Caterson B (2007). Macromolecular organization and in vitro growth characteristics of scaffold-free neocartilage grafts. J. Histochem. Cytochem..

[11] Niemeyer P, Pestka JM, Salzmann GM, Südkamp NP, Schmal H (2012). Influence of cell quality on clinical outcome after autologous chondrocyte implantation. Am. J. Sports Med..

[12] Jonitz-Heincke A (2019). In vitro analysis of the differentiation capacity of postmortally isolated human chondrocytes influenced by different growth factors and oxygen levels. Cartilage..

[13] Brochhausen C (2009). Signalling molecules and growth factors for tissue engineering of cartilage—what can we learn from the growth plate?. J. Tissue Eng. Regen. Med..

[14] Chiu LL, To WT, Lee JM, Waldman SD (2017). Scaffold-free cartilage tissue engineering with a small population of human nasoseptal chondrocytes. Laryngoscope..

[15] Chua KH, Aminuddin BS, Fuzina NH, Ruszymah BH (2007). Basic fibroblast growth factor with human serum supplementation: Enhancement of human chondrocyte proliferation and promotion of cartilage regeneration. Singapore Med J..

[16] Elder BD, Athanasiou KA (2009). Systematic assessment of growth factor treatment on biochemical and biomechanical properties of engineered articular cartilage constructs. Osteoarthr. Cartil..

[17] Huang X, Zhong L, Post JN, Karperien M (2018). Co-treatment of TGF-β3 and BMP7 is superior in stimulating chondrocyte redifferentiation in both hypoxia and normoxia compared to single treatments. Sci. Rep..

[18] Müller S, Lindemann S, Gigout A (2020). Effects of Sprifermin, IGF1, IGF2, BMP7, or CNP on bovine chondrocytes in monolayer and 3D culture. J. Orthop. Res..

[19] Karnieli O (2017). A consensus introduction to serum replacements and serum-free media for cellular therapies. Cytotherapy..

[20] Cuende N, Rasko JE, Koh MB, Dominici M, Ikonomou L (2018). Cell, tissue and gene products with marketing authorization in 2018 worldwide. Cytotherapy..

[21] Mendicino M, Fan Y, Griffin D, Gunter KC, Nichols K (2019). Current state of US Food and Drug Administration regulation for cellular and gene therapy products: Potential cures on the horizon. Cytotherapy..

[22] Neri S (2019). Genetic stability of mesenchymal stromal cells for regenerative medicine applications: A fundamental biosafety aspect. Int. J. Mol. Sci..

[23] Sato Y (2019). Tumorigenicity assessment of cell therapy products: The need for global consensus and points to consider. Cytotherapy..

[24] Teo AQA (2019). Equivalent 10-year outcomes after implantation of autologous bone marrow–derived mesenchymal stem cells versus autologous chondrocyte implantation for chondral defects of the knee. Am. J. Sports Med..

[25] Buckwalter JA, Mankin HJ (1997). Instructional course lectures, The American academy of orthopaedic surgeons-Articular Cartilage. Part II: Degeneration and osteoarthrosis, repair, regeneration, and transplantation. JBJS..

[26] Grose R, Dickson C (2005). Fibroblast growth factor signaling in tumorigenesis. Cytokine Growth Factor Rev..

[27] Inman GJ (2011). Switching TGFβ from a tumor suppressor to a tumor promoter. Curr. Opin. Genet. Dev..

[28] Wallenborn M (2018). Comprehensive high-resolution genomic profiling and cytogenetics of human chondrocyte cultures by GTG-banding, locus-specific FISH, SKY and SNP array. Eur. Cell Mater..

[29] Stumm M (2012). Genomic chondrocyte culture profiling by array-CGH, interphase-FISH and RT-PCR. Osteoarthr. Cartil..

[30] Shafiee A, Kabiri M, Langroudi L, Soleimani M, Ai J (2016). Evaluation and comparison of the in vitro characteristics and chondrogenic capacity of four adult stem/progenitor cells for cartilage cell-based repair. J. Biomed. Mater. Res..

[31] Mumme M (2016). Nasal chondrocyte-based engineered autologous cartilage tissue for repair of articular cartilage defects: an observational first-in-human trial. Lancet.

[32] Chua KH, Aminuddin BS, Fuzina NH, Ruszymah BH (2013). Combination of basic fibroblast growth factor, transforming growth factor beta-2 and human serum enhance human cartilage tissue engineering. Regener. Res..

[33] Neri S, Mariani E, Cattini L, Facchini A (2011). Long-term in vitro expansion of osteoarthritic human articular chondrocytes do not alter genetic stability: A microsatellite instability analysis. J. Cell. Physiol..

[34] Richmon JD (2005). Effect of growth factors on cell proliferation, matrix deposition, and morphology of human nasal septal chondrocytes cultured in monolayer. Laryngoscope..

[35] Wan Kamarul Zaman WS, Makpol S, Sathapan S, Chua KH (2014). Long-term in vitro expansion of human adipose-derived stem cells showed low risk of tumorigenicity. J. Tissue Eng. Regen. Med..

[36] Kang SW, Kim J, Shin DY (2016). Inhibition of senescence and promotion of the proliferation of chondrocytes from articular cartilage by CsA and FK506 involves inhibition of p38MAPK. Mech Ageing Dev..

[37] Tallheden T (2005). Human serum for culture of articular chondrocytes. Cell Transplant..

[38] Sekiya I, Muneta T, Horie M, Koga H (2015). Arthroscopic transplantation of synovial stem cells improves clinical outcomes in knees with cartilage defects. Clin. Orthop. Relat. Res..

[39] Zhou G (2018). In vitro regeneration of patient-specific ear-shaped cartilage and its first clinical application for auricular reconstruction. EBioMedicine..

[40] Vodicka P (2020). Oxidative damage in sporadic colorectal cancer: molecular mapping of base excision repair glycosylases in colorectal cancer patients. Int. J. Mol. Sci..

[41] Pagotto S (2018). Hsa-miR-155-5p drives aneuploidy at early stages of cellular transformation. Oncotarget..

[42] Ciccia A, Elledge SJ (2010). The DNA damage response: Making it safe to play with knives. Mol. Cell.

[43] Ou HL, Schumacher B (2018). DNA damage responses and p53 in the aging process. Blood.

[44] Hartmann A (2003). Recommendations for conducting the in vivo alkaline Comet assay. Mutagenesis.

[45] Darzynkiewicz Z, Huang X, Zhao H (2017). Analysis of cellular DNA content by flow cytometry. Curr. Protoc. Immunol..

[46] Kamil SH (2002). Tissue-engineered human auricular cartilage demonstrates euploidy by flow cytometry. Tissue Eng..

[47] Ogasawara T (2009). Transforming growth factor-β1 in combination with fibroblast growth factor-2 and insulin-like growth factor-I for chondrocyte proliferation culture and cartilage regenerative medicine. Asian J. Oral Maxillofac. Surg..

[48] Schade AE, Fischer M, DeCaprio JA (2019). RB, p130 and p107 differentially repress G1/S and G2/M genes after p53 activation. Nucleic Acids Res..

[49] Dynlacht BD, Flores O, Lees JA, Harlow E (1994). Differential regulation of E2F transactivation by cyclin/cdk2 complexes. Gene Dev..

[50] Engeland K (2018). Cell cycle arrest through indirect transcriptional repression by p53: I have a DREAM. Cell Death Differ..

[51] Molchadsky A, Rivlin N, Brosh R, Rotter V, Sarig R (2010). p53 is balancing development, differentiation and de-differentiation to assure cancer prevention. Carcinogenesis.

[52] Yun MH, Gates PB, Brockes JP (2013). Regulation of p53 is critical for vertebrate limb regeneration. Proc. Natl. Acad. Sci. USA.

[53] Hashimoto S (2009). Role of p53 in human chondrocyte apoptosis in response to shear strain. Arthritis Rheum..

[54] Kim HJ, Park SR, Park HJ, Choi BH, Min BH (2005). Potential predictive markers for proliferative capacity of cultured human articular chondrocytes: PCNA and p21. Artif. Organs..

[55] Cho Y, Gorina S, Jeffrey PD, Pavletich NP (1994). Crystal structure of a p53 tumor suppressor-DNA complex: understanding tumorigenic mutations. Science.

[56] Ognjanovic S, Olivier M, Bergemann TL, Hainaut P (2012). Sarcomas in TP53 germline mutation carriers: A review of the IARC TP53 database. Cancer.

[57] Mantovani F, Collavin L, Del Sal G (2019). Mutant p53 as a guardian of the cancer cell. Cell Death Differ..

[58] Phang BH, Chua HW, Li H, Linn YC, Sabapathy K (2011). Characterization of novel and uncharacterized p53 SNPs in the Chinese population–intron 2 SNP co-segregates with the common codon 72 polymorphism. PLoS ONE.

[59] Brandl A (2010). Influence of the growth factors PDGF-BB, TGF-β1 and bFGF on the replicative aging of human articular chondrocytes during in vitro expansion. J. Orthop. Res..

[60] Williams R (2010). Identification and clonal characterisation of a progenitor cell sub-population in normal human articular cartilage. PLoS ONE.

[61] Cai J (2014). Whole-genome sequencing identifies genetic variances in culture-expanded human mesenchymal stem cells. Stem Cell Rep..

[62] Olive PL, Banáth JP (2006). The comet assay: a method to measure DNA damage in individual cells. Nat. Protoc..

[63] Manthey KC, Rodriguez-Melendez R, Hoi JT, Zempleni J (2006). Riboflavin deficiency causes protein and DNA damage in HepG2 cells, triggering arrest in G1 phase of the cell cycle. J. Nutr. Biochem..

